# White Matter Lesions Are Not Related to **β**-Amyloid Deposition in an Autopsy-Based Study

**DOI:** 10.1155/2011/826862

**Published:** 2011-12-08

**Authors:** Loes C. A. Rutten-Jacobs, Frank-Erik de Leeuw, Lenny Geurts-van Bon, Marije C. Gordinou de Gouberville, Annelieke N. Schepens-Franke, P. Jos Dederen, Wim G. M. Spliet, Pieter Wesseling, Amanda J. Kiliaan

**Affiliations:** ^1^Department of Neurology, Donders Centre for Neuroscience, Medical Centre, Radboud University Nijmegen, 6500 HB Nijmegen, The Netherlands; ^2^Department of Anatomy, Radboud University Nijmegen Medical Centre, P.O. Box 9101, 6500 HB Nijmegen, The Netherlands; ^3^Department of Pathology, University Medical Center Utrecht, 3584 CX Utrecht, The Netherlands; ^4^Department of Pathology, Radboud University Nijmegen Medical Centre, 6500 HB Nijmegen, The Netherlands; ^5^Department of Cognitive Neuroscience, Donders Centre for Neuroscience, Radboud University Nijmegen, 6500 HB Nijmegen, The Netherlands

## Abstract

Population-based studies have investigated the relation between **β**-amyloid levels in cerebrospinal fluid or plasma and white matter lesions (WMLs). However, these circulating levels of **β**-amyloid in cerebrospinal fluid or plasma may not reliably reflect the actual degree of amyloid present in the brain. Therefore, we investigated the relation between WMLs and **β**-amyloid plaques and amyloid angiopathy in brain tissue. WML on MRI or CT were rated in 28 nondemented patients whose neuroimaging was available prior to death. **β**-amyloid in plaques and arterioles were immunohistochemically stained and quantified in postmortem brain necropsies. WMLs were present in 43% of the total population. Both cortex and periventricular region showed no differences for **β**-amyloid deposition in either plaques or blood vessel walls in patients with WMLs compared to those without WMLs. Thus, our results indicate that there is no relation between the degree of WMLs and **β**-amyloid deposition in the brain.

## 1. Introduction

Cerebral white matter lesions (WMLs) are commonly found on cerebral magnetic resonance imaging (MRI) and computerized tomography (CT) in elderly subjects [1, 2]. The frequency of WML increases with age [3], and they are related to both cognitive impairment [4] and Alzheimer's disease (AD) [5].

From an etiological perspective, heterogeneity exists with respect to the presence of WML: on the one hand, they are related to cardiovascular risk factors, including hypertension and atherosclerosis [6–8], but on the other hand, they are abundantly present in patients with an underlying amyloid pathology such as cerebral amyloid angiopathy (CAA) [9].

On a population level, there is also evidence for such heterogeneity since a relation between WMLs on MRI and levels of *β*-amyloid in cerebrospinal fluid or plasma has been established [3, 10]. However, a recent study did not find any association between *β*-amyloid in cerebrospinal fluid and WML [11]. Furthermore, there is no confirmation of *β*-amyloid pathology in brain tissue in these particular studies. There are a few neuropathological studies which encountered a relation between the degree of CAA, *β*-amyloid plaques, and the severity of WML in patients with clinically confirmed AD, albeit that both the amyloid and white matter pathology were assessed on a microscopic level and not with neuroimaging [12, 13].

To investigate the relation between cerebral amyloid pathology within white matter lesions and the degree of white matter lesions on neuroimaging, one should preferably look for amyloid pathology in a sample of the white matter. This may have important implications for our understanding of the etiology and found heterogeneity of presumed vascular white matter lesions and provide a model for interaction between vascular and amyloid pathology in the pathophysiology of WMLs. To our best knowledge, there are no studies relating the presence of WMLs on neuroimaging with neuropathologically assessed amyloid pathology.

We therefore investigated the relation between the degree of *β*-amyloid deposition in microvessels and plaques in necropsies from the cerebral white matter and WMLs and WMLs assessed on MRI in an autopsy-based study of nondemented patients.

## 2. Materials and Methods

### 2.1. Study Population

The study population consisted of 28 consecutive patients without any age restriction from whom neuroimaging was available on average 2.8 months prior to death. No significant difference exists in time of imaging prior to death between the WMLs groups. Neuroimaging was performed for routine investigations because of a suspicion of a neurological disorder not related to dementia (minor stroke, minor traumatic brain injury, and exclusion of brain tumor). None of the patients suffered from dementia according to NINDS-AIREN [14] and NINCDS-ADRDA [15] criteria and all of them died of noncerebral or nonneurodegenerative causes. Autopsy consent was obtained from the next of kin. The study was approved by the Medical Ethics Committee of University Medical Center Utrecht, the Netherlands.

### 2.2. Tissue Samples

Within 16 hours after death, necropsies were taken from the frontal lobe region of the hemisphere which was predominantly affected by the WMLs. When trauma or stroke occurred in this hemisphere, the necropsy was taken from the other, unaffected hemisphere. Samples, each about 2 cm^3^ in size, were taken from the frontal grey matter and the underlying periventricular region in a standardized way, guided by the obtained CT or MRI scan. The periventricular region was defined as the region from the ventricle wall extending 1 cm into the white matter.

Tissue was fixed in 4% paraformaldehyde in 0.1 M phosphate buffer (pH 7.4) and embedded in paraffin according to a standard protocol.

### 2.3. *β*-Amyloid Immunohistochemistry

*β*-amyloid was demonstrated immunohistochemically on 4 *μ*m thin paraffin sections using monoclonal antibodies against the *β*_A4_-amyloid (1 : 100; clone 6F/3D, DAKO M0872, Glostrup, Denmark).

To determine the amount of *β*-amyloid, sections were digitalized and quantified, using a Zeiss Axioskop microscope equipped with hardware and software of Microbrightfield, (Williston, USA). Quantitative analyses were performed with a computer-assisted analysis system (Stereo Investigator) using Cavalieri's probe. Total *β*-amyloid staining in both plaques and cerebral blood vessel walls was measured by two independent raters in a random 25 × 10^6^ *μ*m^2^ cortex and periventricular white matter area. The *β*-amyloid deposition in plaques was expressed as the area of *β*-amyloid positive plaques divided by the total area measured, times 100 in order to obtain a percentage of the affected area. Subsequently, *β*-amyloid deposition in cerebral blood vessels was quantified in a similar manner. Immunohistochemistry and determination of *β*-amyloid positivity was measured without knowledge of rating of the WMLs or any other clinical data.

### 2.4. Rating of White Matter Lesions

WMLs were rated with the age-related white matter changes (ARWMCs) rating scale by one single experienced rater (FEdL) [16]. The use of this rating scale has the advantage that it can be used for rating WMLs on both CT and MRI with high inter- and intrarater agreement. In short, WMLs were defined as ill-defined hyperintensities ≥5 mm on MRI images or as ill-defined and moderately hypodense areas of ≥5 mm on CT images. They were rated on a four-point scale in five different regions in the left and right hemisphere separately: (1) the frontal area, (2) the parieto-occipital area, (3) the temporal area, (4) the infratentorial area, and (5) the basal ganglia. WMLs were scored semiquantitatively as 0 (no WMLs), 1 (single or multiple focal lesions ≥5 mm), 2 (beginning of confluence of lesions) or 3 (confluent WMLs).

Total degree of WMLs was calculated by adding the region-specific scores of both hemispheres (range 0–30).

### 2.5. Statistical Analysis

We compared the *β*-amyloid load in plaques and in cerebral blood vessels in patients with WMLs (ARWMC > 0) or without WMLs (ARWMC = 0) with the Mann-Whitney *U* test because of a nonparametric distribution of the amyloid deposition. To identify differences in population characteristics between groups, the Mann-Whitney *U* test and Chi-square test were used.

To assess whether any observed differences were influenced by age, partial Spearman's correlations were calculated between WMLs and *β*-amyloid, adjusting for age and gender.

## 3. Results

Table 1 shows the characteristics of the study population. Mean age was 63.1 yrs (SD 15.9 yrs; range 18–84 yrs); 39% was female. WMLs were present in 43% of the total population. Mean coverage of the area by *β*-amyloid plaques was 0.08% (range 0.0 to 1.02%) in the cortex and 0.0003% (range 0.0 to 0.003%) in the periventricular white matter region. Mean coverage of the area by vascular amyloid was 0.005% (range 0.0 to 0.040%) in the cortex, with no amyloid found in vessels in the periventricular white matter.

**Figure 1:**
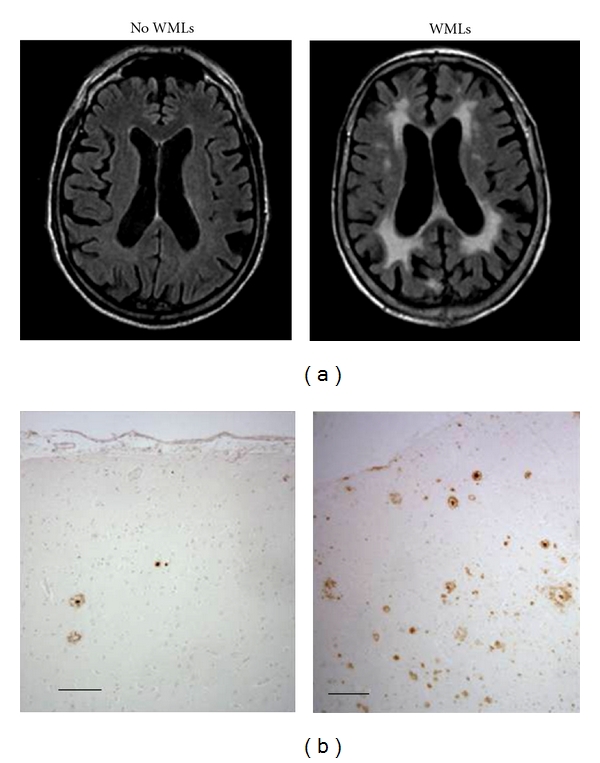
(a) MRI scans with and without white matter lesions and (b) corresponding *β*-amyloid pathology in the cortex. Bar = 100 *μ*m.

Patients with WMLs had significantly more cortical *β*-amyloid plaques than those without WMLs (0.17% (SD 0.33) versus 0.01% (SD 0.03); *P* < 0.05, Figure 1). However, both groups differed in age (Table 1). Therefore, we assessed the influence of age on a possible correlation between cortical *β*-amyloid coverage and WMLs. After adjusting by age, no correlation could be found between cortical *β*-amyloid coverage and WMLs (crude Spearman's Rho correlation coefficient = 0.41, *P* < 0.05, age-adjusted correlation coefficient = 0.17, *P* = 0.40).

The amount of *β*-amyloid plaques in the periventricular area and vascular amyloid in both the cortical and periventricular area did not differ between the WMLs groups also not after adjustment for age and gender.

## 4. Discussion

We found no association between WMLs and *β*-amyloid deposition in the brain of elderly nondemented persons.

To our best knowledge, this study is the first to investigate a possible relation between amyloid pathology and the presence of WMLs on MRI in nondemented elderly by directly assessing the load of *β*-amyloid in the brain, rather than an indirect measure of amyloid pathology (cerebral spinal fluid or plasma levels).

In accordance with a recent study by Jonsson et al. [11], our results do not indicate that *β*-amyloid and WMLs are related.

Our results are not completely in agreement with a study by Stenset et al. [10]. They investigated whether WMLs lesions were inversely related with CSF A*β*42, following the rationale that CSF A*β*42 reduction is seen in AD due to deposition in *β*-amyloid plaques. They found that WMLs and hypercholesterolemia explained only 9% of the variability in CSF A*β*42, suggesting that other factors must play a role in the development of white matter lesions. Having hypercholesterolemia raised the probability of low A*β*42 levels by 20%, while for each point increase in WMLs score (same scale as used in our study), the probability of low CSF A*β*42 levels only increased by 3%. In contrast to our findings, A*β*42 levels did not correlate with age. However, it remains controversial whether amyloid markers either in CSF or blood plasma reflect actual amyloid pathology in the brain [17–19].

A possible weakness of our study was the small number of patients. Since not even a trend towards a relation between amyloid pathology in the brain and WMLs on neuroimaging could be detected in our sample, we expect this relation to be weak, especially compared to the strength of the association with other common factors like age. However, one has to keep in mind that a type II error may have lead to false negative findings in our study.

Neuroimaging was performed on average 3 months prior to death. WMLs might have progressed in this limited time interval, but this is very unlikely, as the progression rate of WMLs is known to be very slow [3, 20].

Theoretically, staining of the tissue might vary by reduced immunoreactivity due to different postmortem times, but in this study, there were no differences in postmortem time between groups (data not shown).

Before clinical signs of AD are perceptible, both WMLs assessed on MRI and *β*-amyloid plaques in the cortex are already present [1, 21] and increase with age. The presence of white matter lesions in AD is often seen; moreover, these WMLs and AD share some risk factors, wit hage being the most important. Consequently, coexisting WMLs and AD results in more severe cognitive impairment, either by direct damage of the neural pathway, or indirect by worsening the impact of AD pathology [22].

As hypothesized by others, this suggests that cerebrovascular disease may be in the causal pathway for development of AD or interacts synergistically with AD pathology.

The presence of *β*-amyloid plaques has been considered the defining characteristic of AD, and it forms the core of the most studied theoretical framework for AD: the amyloid cascade hypothesis [23].

On the other hand, WMLs might be more supportive of the vascular hypothesis [24, 25]. WMLs are thought to have a vascular origin, because vascular risk factors, especially hypertension, are related to the presence of these lesions [8]. Furthermore, evidence suggests that white matter is highly vulnerable to hypoxia due to vascular dysfunction [26, 27]. In the last decade, several large population-based studies have highlighted the important contribution of vascular risk factors in AD [28–30].

As a result, some researchers are in favor of the amyloid cascade hypothesis, while others believe in the vascular hypothesis. However, the association between vascular risk factors and AD does not rule out degenerative mechanisms underlying pure AD. It can be hypothesized that vascular and degenerative mechanisms actually develop in parallel. Vascular brain injury could act additively or synergistically with concomitant

AD pathology to produce more severe cognitive dysfunction than either process alone [31].

To conclude, our results show no association between WMLs and *β*-amyloid load in the brain of elderly nondemented persons. Although they might have causal factors in common, WMLs and *β*-amyloid load in the brain could represent separate pathways to AD.

Further research is necessary to clarify these complex relationships. Prospective, multimodal imaging studies in disease-free individuals that both visualize the degree of amyloid pathology (e.g., with PET-PIB) [32] and the degree of WMLs are needed to unravel the chain of events from amyloid pathology and white matter lesions and the attendant cognitive decline [33].

## Figures and Tables

**Table 1:** Characteristics of the study population.

	Total (*n* = 28)	no WMLs (*n* = 16)	WMLs (*n* = 12)
Age, years	63.1 (15.9)	56.8 (13.6)	71.6 (15.3)^#^
Women, %	39.3	18.8	66.7*
Hypertension, %	46.2	33.3	63.6
Diabetes mellitus, %	11.1	13.3	8.3
Cerebral infarct, %	15.4	14.3	16.7
Myocardial infarction, %	7.4	6.7	8.3
Median ARWMC	0	0	8
Ventricle-to-brain ratio	0.36 (0.06)	0.36 (0.06)	0.36 (0.05)

Values are unadjusted means (SD) or percentages.

^∗#^Mean or percentage is significantly different from people with no WMLs, *P* < 0.05 and *P* < 0.01, respectively.
